# Rumination in the Human Subject

**Published:** 1845-05

**Authors:** Robert Read

**Affiliations:** Veterinary Surgeon, Crediton


*Rumination in the Human Subject.*—By Robert Read, Veterinary Surgeon, Crediton.—F. L———, aged twenty-four, an idiot and an inmate of the Crediton Union Workhouse, possesses this singular propensity, so foreign to man. He rejects, if offered to him, every kind of compact animal food, but eagerly devours, with imperfect mastication, farinaceous and other vegetable substances, more especially boiled potatoes, of which he is very fond; in fact, he subsists almost wholly on a vegetable diet, with the exception, occasionally, of a little broth. Animal food, when offered to him, is thrown away with displeasure. After he has made a repast, he will sometimes, in from ten to twenty minutes, begin to regurgitate and to remasticate the food over again; at other times, an hour or even more will pass away before any attempt to ruminate is made. The act with him is perfectly voluntary, as he can command it or suspend the same at his will or pleasure. The act of regurgitating his food does not produce any uneasy or disquiet feeling, neither does it create the least semblance to vomition, in which the efforts are painful and convulsive. His position is generally, when in the act of chewing his food over again, fixed, with his back against a wall; he then raises his shoulders, contracts the abdominal muscles, so as to press against the stomach, and makes an expiration. Thus the food is again, with every appearance of ease, returned to the mouth for a second mastication. This act of rumination I have no doubt he continues until he has brought all the solid parts of his food into a semi-fluid state. He seems to enjoy the process of rumination more than his meal; as, in the latter, it is hastily devoured, whilst in the former he seems to be quiet, calm, and pleased. Excitement or alarm, or any mistrust or attention to him, will make him suspend the act, but as soon as such causes are absent, he again returns to rumination, chewing the food over again. Rumination is peculiar only to animals which are classed under the order ruminantia, herbivora, possessing a plurality of stomachs. In the ox, the food does not return again to the same receptacle from which it is regurgitated. Rumination therefore in man must differ in this respect, as it is highly improbable that there is more than one stomach in man-ruminants; but the comparative repose and quiet in each is not dissimilar during the act. The reason, speaking physiologically, why this man-ruminant refuses animal food, may be from the change effected in the stomach during digestion, whereby something nauseous is eliminated, rendering the pellet returned to his mouth disagreeable to the taste, whilst a diet purely vegetable during the digestive process may generate a saccharine agent, and thus render its rumination more pleasing to him. Some cases are recorded in which the act of rumination in man is one of a painful effort, attended with spasmodic and other convulsive signs, effusion of tears, and the eyes bursting from their orbit. It is not the case with the one I have detailed. Many of the readers of *The Lancet* can now theorize on the subject of rumination in man; I have supplied the outlines of the case as near as I can. Comparative anatomists agree that all ruminant animals are quadrupeds, "viviparous," and have four stomachs; but in the case of this poor idiot there is an exception to the general rule, which can only be determined by an examination after death. Diversity exists as to the relative quality of the food for rumination in man and the ox; the latter scarcely, if ever, returns farinaceous food or boiled bulbous roots, as by the first mastication and the action of the paunch they are reduced sufficiently to a pultaceous mass; the comminuting process is only required the second time when staminaceous food forms their diet, whilst in man who ruminates, animal food is rejected, and bulbous food is chosen for remastication, as is clearly exemplified in this case. The contractile power of his stomach seems to be very great, as when teased or irritated he will eject the fluid contents of his stomach to a considerable distance through his mouth. In so doing he must make the œsophagus a passive tube, as no resistance seems to be offered by it. In concluding this most interesting case, I can only say it is surprising that it has remained so long unheeded and unrecorded in any medical journal, although seen by so many medical men. I beg pardon of them for publishing it, but must say that the ruminating part of the question is more applicable to my province and practice.—*Lancet.*

